# Urinary based biomarkers identification and genetic profiling in Parkinson’s disease: a systematic review of metabolomic studies

**DOI:** 10.3389/fbinf.2025.1513790

**Published:** 2025-03-10

**Authors:** Neetu Rani Dhiman, Surbhi Singh, Royana Singh, Anand Kumar, Varun Kumar Singh, Abhishek Pathak, Rameshwar Nath Chaurasia, Vijay Nath Mishra, Niraj Kumar Srivastava, Swati Sahu, Nikhil Pandey, Deepika Joshi

**Affiliations:** ^1^ Department of Neurology, Institute of Medical Sciences, Banaras Hindu University, Varanasi, Uttar Pradesh, India; ^2^ Department of Anatomy, Institute of Medical Sciences, Banaras Hindu University, Varanasi, Uttar Pradesh, India

**Keywords:** Parkinson’s disease, metabolomics, review, genetic profile, urine based diagnostics

## Abstract

**Background:**

Parkinson’s disease is a complex, age-related, neurodegenerative disease associated with dopamine deficiency and both motor and nonmotor deficits. Therapeutic pathways remain challenging in Parkinson’s disease due to the low accuracy of early diagnosis, the difficulty in monitoring disease progression, and the limited availability of treatment options.

**Objectives:**

Few data are present to identify urinary biomarkers for various ailments, potentially aiding in the diagnosis and tracking of illness progression in individuals with Parkinson’s disease. Thus, the analysis of urinary metabolomic biomarkers (UMB) for early and mid-stage idiopathic Parkinson’s disease (IPD) is the main goal of this systematic review.

**Methods:**

For this study, six electronic databases were searched for articles published up to 23 February 2024: PubMed, Ovid Medline, Embase, Scopus, Science Direct, and Cochrane. 5,377 articles were found and 40 articles were screened as per the eligibility criteria. Out of these, 7 controlled studies were selected for this review. Genetic profiling for gene function and biomarker interactions between urinary biomarkers was conducted using the STRING and Cytoscape database.

**Results:**

A total of 40 metabolites were identified to be related to the early and mid-stage of the disease pathology out of which three metabolites, acetyl phenylalanine (a subtype of phenylalanine), tyrosine and kynurenine were common and most significant in three studies. These metabolites cause impaired dopamine synthesis along with mitochondrial disturbances and brain energy metabolic disturbances which are considered responsible for neurodegenerative disorders. Furoglycine, Cortisol, Hydroxyphenylacetic acid, Glycine, Tiglyglycine, Aminobutyric acid, Hydroxyprogesterone, Phenylacetylglutamine, and Dihydrocortisol were also found commonly dysregulated in two of the total 7 studies. 158 genes were found which are responsible for the occurrence of PD and metabolic regulation of the corresponding biomarkers from our study.

**Conclusion:**

The current review identified acetyl phenylalanine (a subtype of phenylalanine), tyrosine and kynurenine as potential urinary metabolomic biomarkers for diagnosing PD and identifying disease progression.

## Introduction

With a frequency of 1% in those over 60, Parkinson’s disease (PD) is one of the most prevalent neurodegenerative illnesses (De Lau and Breteler, 2006). The distinctive motor symptoms, such as hypokinesia, postural instability, stiffness, and resting tremor, are brought on by a lack of dopaminergic input to the striatum when the degeneration of dopaminergic neurons in the substantia nigra reaches 50%–60%. These clinical signs form the bulk of the diagnostic (Rocca, 2005). The diagnosis of Parkinson’s disease (PD) is now made mainly on clinical observation, although early identification is difficult due to the vast variety of symptoms, many of which are shared by other neurodegenerative diseases. Since the symptoms of PD match those of other neurodegenerative diseases, clinical diagnosis of PD is not always accurate (between 75% and 90%) but blood or laboratory tests are not yet available to reliably diagnose Parkinson’s disease in clinical settings. Because there are few disease-modifying treatments that can stop or slow the development of the disease, the condition is often managed symptomatically (Alexander, 2004).

Finding biomarkers that can more precisely anticipate the illness is, therefore, necessary in order to advance the development of therapies and enable more accurate disease monitoring, both of which would ultimately improve the prognosis of PD patients. A technique now required in the evaluation and validation of PD is the use of biomarkers for early diagnosis and disease progression prediction (Jankovic, 2008). Metabolomic studies in PD have improved knowledge of the underlying molecular pathways and led to the development of biomarkers for early diagnosis. Additionally, it has the ability to differentiate various metabolite variants and find genetic changes that could be important functionally. Metabolomics-based techniques also deliver objective, high output, valuable data of a variety of good consistency metabolites. They form an attractive method for identifying possible biomarkers in Parkinson’s disease (PD). Changes in the profiles of urine metabolites may signal the early stages of Parkinson’s disease. Given that urine collects non-invasive samples and contains the majority of the metabolic bi-products of the body, it is always a “favoured” marker source for disease study (Bouatra et al., 2013; Luan et al., 2015a). Urine holds an important part of acting as a biomarker in neuro-degenerative conditions as it is available non-invasively and it also mirrors biochemical changes in blood (Eller and Williams, 2009). Urinary 8-hydroxy-2’ (8-OHdG), a valuable bi-product in oxidative damage of DNA, is correlated with PD progression. Urine is a significant biofluid that is rich in metabolites that can be used as biomarkers to diagnose neurological disorders. It also contains the metabolic end products and is the most convenient way of sampling than blood and CSF (Bai et al., 2021). Changes in urine composition may be more helpful than those in serum (Bolner et al., 2011).

Presently, no biomedical tests are available to forecast the course of PD illness, despite the fact that it is widely acknowledged that such prediction tools are essential to the field. Despite the fact that there are substantial data on other body fluids, the non-invasive nature, abundance, stability, and rich metabolomic and proteomic content (Luan et al., 2015b) make urine a highly valuable fluid for PD biomarker discovery, potentially complementing or even surpassing other fluids traditionally studied for this purpose. The potential for discovering novel biomarkers in urine could significantly improve early diagnosis and monitoring of Parkinson’s disease. Determining prospective urine-based biomarkers in the early and mid-stage idiopathic PD (IPD), utilizing a metabolomic platform is the goal of this systematic review, which intends to increase the precision of early diagnosis and progression of PD-prediction.

## Materials and methods

The preferred reporting items for systematic reviews and meta-analyses (PRISMA) checklist have been incorporated to write this systematic review (Page et al., 2021).

### Search strategy for literature

A thorough review of literature was done using an extensive exploration employing keywords and subject headings, from which a list of potential biomarkers could be created. Six electronic databases were searched for articles published up to December 2023: PubMed, Ovid Medline, Embase, Scopus, Science Direct, and Cochrane. The search criteria included “Parkinson,” “Parkinson’s Disease,” “metabolites,” “metabolomics,” “metabolic,” “metabolome,” “biomarkers,” “urine-based”, “urinary” and “human” in combination. Terms were looked for using Boolean search in titles, abstracts, and MeSH keywords to provide a specific search result.

### Eligibility criteria for studies

By comparing the articles to the inclusion and exclusion criteria, the eligibility of the articles was established. Articles that did not fit the requirements for inclusion were rejected. A second round of screening was carried out using the full-text content of the articles for the final shortlisting of the studies that met the inclusion criteria.

#### Inclusion criteria

Studies were searched from inception to 23.02.2024. Controlled studies focussing on urine based metabolomic analysis in early and mid-stage PD patients were selected. English language studies were included.

#### Exclusion criteria

Non-research articles like systematic reviews, interventional studies, letter to the editor, narrative reviews were excluded. Proteome and genome based studies with no control group were also excluded.

### Screening and data extraction process

Two reviewers (NRD & DJ) searched and assessed for the eligible studies. They extracted the important data and mentioned in Table 1, based on the listed eligibility criteria. Any disagreement was resolved after a discussion with the third reviewer (SS). Each separately extracted the data in compliance with the study’s methodological characteristics, interventions, participants, outcomes, and conclusions. PRISMA 2020 flowchart outlined the complete selection process.

**TABLE 1:** Description of the studies.

Authors and year	No. of subjects	Country	Sample	Method of metabolomic analysis	Main finding/biomarkers
Chung et al. (2023) (Profiling analysis of Tryptophan metabolites in the urine of patients with Parkinson’s disease using LCMS/MS)	41 PD patients (16-M, 25-F)	Korea	Urine sample	LC-MS/MS	Indole-3-acetic-acid
Wichit et al. (2021) Monoamine levels and Parkinsons’s disease progression: evidence from a High Performance Liquid Chromatography study	24 early PD (M = 18, F = 6) and 16 late PD (M = 9, F = 7)	Thailand	Urine samples	HP-LC	Homovanillic acid (in dopaminergic system) and 5-hydroxyindoleacetic acid (in serotonergic system)
Kumari et al. (2020a) (Identification of potential urine biomarkers in idiopathic Parkinson’s disease using NMR)	100 PD with 62 in early stage (78-M, 22-F)	India	Urine sample	High resolution nuclear magnetic resonance spectroscopic Analysis	Ornithine, tryptophan, phenylalanine, isoleucine, β-hydroxybutyrat, tyrosine and succinate
Bai et al. (2021) (Urinary Kynurenine as a biomarker for Parkinson’s disease)	41 early PD patients 24-male 17-female	China	Urine sample	ELISA to identify KYN levels in urine	Kynurenine
Luan et al. (2015a) (LC−MS-Based Urinary Metabolite Signatures in Idiopathic Parkinson’s Disease)	Total 106 (66-M, 40-F) idiopathic early (sample collected on week 1), 93 (56-M, 37-F) mid-stage (sample collected on week 16) and 98 (54-M, 44-F) late-stage (sample collected on week 32) PD patients	China	Urine sample	High performance LC-MS analysis @45 metabolites were profiled for identification of PD	hydroxyprogesterone, cortisol, tryptamine, 5-hydroxytryptophan, indolelactic acid, kynurenine, hydroxyphenylacetylglycine, phenylacetylglycine, furoglycine, tiglyglycine, glycine, phenylacetylglutamine, acetylphenylalanine, phenylacetic acid, tyrosine, hydroxyphenylacetic acid, glutamyltyrosine, acetyltyrosine, 5,6-dihydroxyindole, deoxyinosine, malonylcarnitine, urocanic acid, spermidine, naphthol, aminobutyric acid, trimethylamine N-oxide
Luan et al. (2015b) (Comprehensive urinary metabolomic profiling and identification of potential noninvasive marker for idiopathic Parkinson’s disease)	14 early (6-M, 8-F) and 59 (41-M, 18-F) mid-stage PD and 19 (8-M, 11-F) late-stage patients	China	Urine sample	LC-MS and GC-MS	Acetyl phenylalanine, kynurenine, hydroxytryptophan, furoylglycine, tyrosine/hydroxyphenylacetic acid, cortisol, glycine, aminobutyric acid, tiglylglycine, hydroxybenzoic acid, hydroxyprogesterone, xanthurenic acid, isoleucine, leucine, alanine, dihydrocortisol phenylacetylglutamine, and phenylalanine
Luan et al. (2015c) (Elevated excretion of biopyrrin as new marker for idiopathic Parkinson’s disease)	14 early (6-M, 8-F) and 59 (41-M, 18-F) mid-stage and 19 (8-M, 11-F) late-stage patients	China	Urine sample	LC-MS analysis and ELISA quantification	Biopyrrin

PD, Parkinson’s Disease; M, male; F, female; LC-MS, liquid chromatography mass spectrophotometry; LC-MS/MS, liquid chromatography tandem mass spectrophotometry; HP-LC, high performance liquid chromatography; GC-MS, gas chromatography mass spectrophotometry.

### Quality assessment

The case-control quality evaluation tool from the National Heart, Lung, and Blood Institute (NHLBI) was used to evaluate the study’s overall quality (National Heart et al., 2013). NHLBI developed a set of tailored quality assessment tools in 2013 to assist reviewers in focussing on concepts that are key to a study’s internal validity. The tools are specific to certain study designs and tested for potential flaws in study methods or implementation. Based on the study design (case-control study), this quality assessment tool was used. The study quality was evaluated by two authors (NRD and NP) separately and was rated as “yes,” “no,” or “not applicable.” A study is considered of “Good” quality if it meets all or most of the specified criteria, with minimal risk of bias. A study classified as “Fair” quality does not meet the specified criteria’s and suggests some risk of bias but not enough to completely undermine the results. A “Poor” quality study has significant methodological flaws that increase the risk of bias and limit the validity of its findings. A third reviewer (SS) was brought in to help address any disputes or issues.

### Data analysis

#### Reproducibility of results

This was solely done on study and patient characteristics and urinary metabolomic biomarkers (UMB).

#### Gene search for PD

To identify genes associated with Parkinson’s disease (PD) and its potential biomarkers, a comprehensive search was performed using the DisGeNET database (v7.0). DisGeNET is a widely recognized open-access platform that provides insights into the genetic underpinnings of human diseases and their phenotypic traits (Piñero et al., 2017). For this study, the dataset corresponding to Parkinson’s disease (C0030567) was selected. A systematic search was conducted to extract the genes linked to PD. In this analysis, two key metrics were introduced to prioritize the genes: the Disease Specificity Index (DSI) and the Disease Pleiotropy Index (DPI). The DSI ranges from 0 to 1, where a score closer to 1 indicates that the gene is highly specific to a single disease. Genes with a DSI of 1 were selected as the most specific to Parkinson’s disease. Gene Network (DisGeNET) database (C0030567) consists of 685 unique PD-related genes. Among these genes, we selected those with a gene-disease-association score greater than or equal to 0.1 (Score-GDA ≥ 0.1), yielding 169 genes. These genes are involved in various functions and related to urinary biomarkers.

#### Protein-protein interaction (PPI) network construction

The genes identified from DisGeNET were used to construct protein-protein interaction (PPI) networks using the STRING database (version 11.5) (Szklarczyk et al., 2023). STRING integrates various sources of interaction data, including experimental studies, computational predictions, and curated biological pathways. The STRING web platform was utilized to filter interactions, selecting those with a confidence score greater than 0.4 and a p-value less than 0.05, ensuring statistical significance. The STRING platform automatically evaluates whether the observed interactions in the network are significantly more than expected in a random set of proteins of similar size.

Both direct and indirect interactions were included in the network.

#### Network analysis and visualization

The constructed PPI networks were imported into CYTOSCAPE (version 3.8.2) (Otasek et al., 2019), a powerful tool designed for the visualization and for analysis of complex biological networks and same confidence score, STRING was selected. In CYTOSCAPE, the networks underwent topological analysis to identify key nodes, which are hypothesized to play critical roles in the pathology of Parkinson’s disease.

#### Identification of hub genes

Hub genes, which are highly interconnected within the PPI network, were identified using the CytoHubba plugin (version 0.1) (Chin et al., 2014) in Cytoscape. These hub genes are of particular interest due to their central roles in maintaining the network structure and regulating essential biological processes. Identifying such hub genes is crucial, as they may serve as key molecular drivers in the development and progression of Parkinson’s disease.

#### Meta-analysis framework

To conduct a meta-analysis, studies related to the identified hub genes and their associations with Parkinson’s disease were systematically reviewed. Data were extracted from multiple independent studies that reported on the genetic, transcriptomic, and proteomic alterations linked to PD. The pooled data were statistically analyzed to quantify the overall effect size and assess the consistency of gene associations across studies. By integrating data from DisGeNET, STRING, and Cytoscape, this methodological approach provided a comprehensive analysis of the genetic and protein interaction networks associated with Parkinson’s disease, enabling the identification of potential molecular targets for therapeutic interventions. This meta-analytic framework aimed to strengthen the validity of gene associations and uncover novel biomarkers for Parkinson’s disease diagnosis and treatment.

## Results

### Study selection on literature search

**FIGURE 1:**
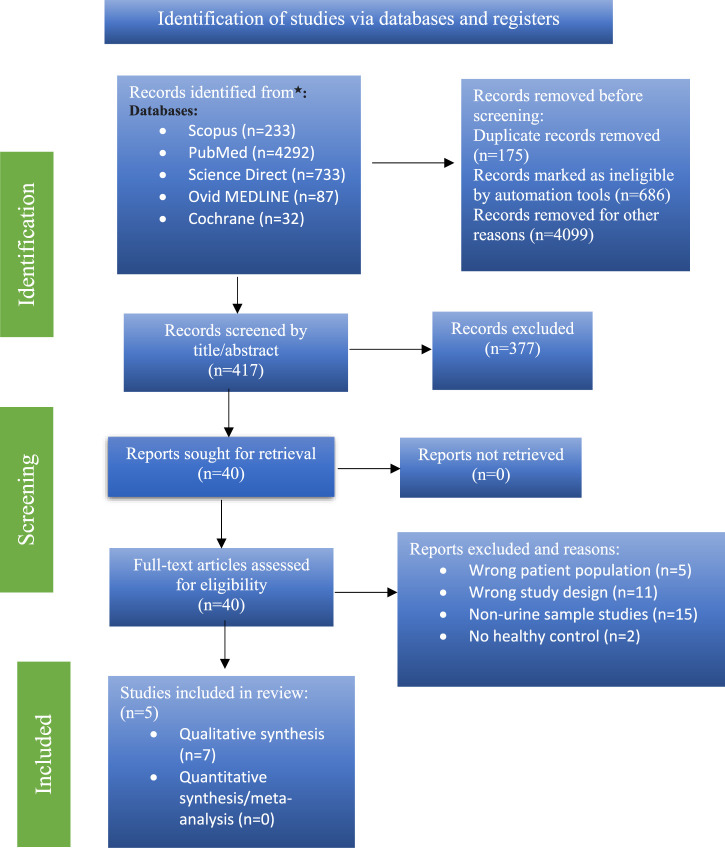
PRISMA flow chart of the study selection process.

5,377 items were found during a primary literature search. The qualifying criteria were followed in the screening of 40 articles. Seven (Luan et al., 2015a; Bai et al., 2021; Luan et al., 2015b; Chung et al., 2023; Wichit et al., 2021; Kumari et al., 2020a; Luan et al., 2015c) of these studies satisfied the requirements for inclusion in the review. A PRISMA flow chart is shown for the search parameters, study selection, and study exclusion in Figure 1.

### Studies description

This review was based solely on studies with urinary metabolomic biomarkers in early and mid-onset PD. Out of total 40 studies on PD biomarkers, only 7 studies were included based on selection criteria. All studies passed the NIH quality assessment evaluation and were of “fair” quality (Table 2).

**TABLE 2:** The NIH quality assessment method was used to evaluate the risk or bias of all included case-control studies (n = 5).

Criteria	Luan et al. (2015a)	Luan et al. (2015b)	Luan et al. (2015)	Bai et al. (2021)	Kumari et al. (2020a)	Chung et al. (2023)	Chung et al. (2023); Wichit et al. (2021)
1. Was there search question or objective in this paper clearly stated and appropriate?	Y	Y	Y	Y	Y	Y	Y
2. Was the study population clearly specified and defined?	Y	Y	Y	Y	Y	Y	N
3. Did the authors include a sample size justification?	N	N	N	N	N	N	N
4. Were controls selected or recruited from the same or similar population that gave rise to the cases (including the same timeframe)?	Y	N	Y	Y	Y	Y	Y
5. Were the definitions, inclusion and exclusion criteria, algorithms or processes used to identify or select cases and controls valid, reliable, and implemented consistently across all study participants?	Y	Y	Y	Y	Y	Y	Y
6. Were the cases clearly defined and differentiated from controls?	Y	Y	Y	Y	Y	Y	N
7. If less than100% of eligible cases and/or controls were selected for the study, were the cases and/or controls randomly selected from those eligible?	NA	NA	NA	NA	NA	Y	NA
8. Was there use of concurrent controls?	Y	Y	Y	Y	Y	Y	Y
9. Were the investigators able to confirm that the exposure/risk occurred prior to the development of the condition or event that defined a participant as a case?	Y	Y	Y	Y	Y	Y	Y
10. Were the measures of exposure/risk clearly defined, valid, reliable, and implemented consistently (including the same time period) across all study participants?	Y	Y	Y	Y	Y	Y	Y
11. Were the assessors of exposure/risk blinded to the case or control status of participants?	N	N	N	N	N	N	N
12. Were key potential confounding variables measured and adjusted statistically in the analyses? If matching was used, did the investigators account for matching during study analysis?	N	Y	N	Y	Y	Y	Y
Quality	Fair	Fair	Fair	Fair	Fair	Good	Fair

Quality was rated as poor (0–4 out of 12 questions), fair (5–10 out of 12 questions), or good (11–12 out of 12 questions); Y, yes; N, No and NA, not applicable.

**FIGURE 2:**
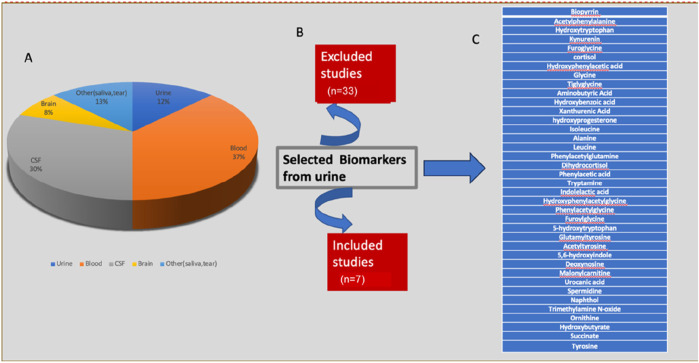
An overview of the different types of samples used in various studies and the identitification of **(A)** all replicated UMB, **(B)** the total number of studies that were included and excluded, and **(C)** Urinary biomarkers identified.

Total number of PD patients in all studies who participated was 512 and total number of healthy age and gender matched control volunteers was 385. 321 were male and 191 were female patients. A summary of the type of samples and identified UMB is described in Figure 2. P-value (mann-whitney U test), Mean decrease in accuracy (MDA), Fold change (FC) value more >1.2 indicating upregulation in PD, and Area under the ROC curve (AUC) value (determined by Youden index) was kept at 0.59 to 0.95 indicating potential metabolites (Supplementary Table S1).

### Reproducibility in the findings of all studies

All studies focussed on identifying urinary biomarkers for IPD. All three studies conducted by Hemi Luan et al., and Chung SH et al. used LC-MS to identify metabolites. LC-MS is faster, highly sensitive and capable of detecting low-abundance molecules than Nuclear Magnetic Resonance. Reproducible findings about the metabolic alterations in PD in all three studies contribute to the understanding that oxidative stress, energy metabolism and neurotransmitter metabolism pathways act as a target for biomarkers. Reproducibility was also indicated by the occurrence of common UMB in these studies. The availability of similar UMB with matched Fold Change (FC) could determine a potential urinary biomarker in early (acetylphenylalanine, hydroxytryptophan, tyrosine, kynurenine, cortisol, tiglyglycine, phenylacetylglutamine, kynurenine) and mid-onset (hydroxytryptophan, tyrosine/hydroxyphenylacetic acid, phenylacetylglutamine) PD.

### Gene search for Parkinson’s disease

From total 685 genes, 169 genes were selected from the Parkinson’s disease (C0030567) dataset in DisGeNET, prioritized based on their Disease Specificity Index (DSI). Genes with a DSI closest to 1, indicating higher specificity to Parkinson’s disease, were given preference for further analysis.

### Protein-protein interaction (PPI) network construction

**FIGURE 3:**
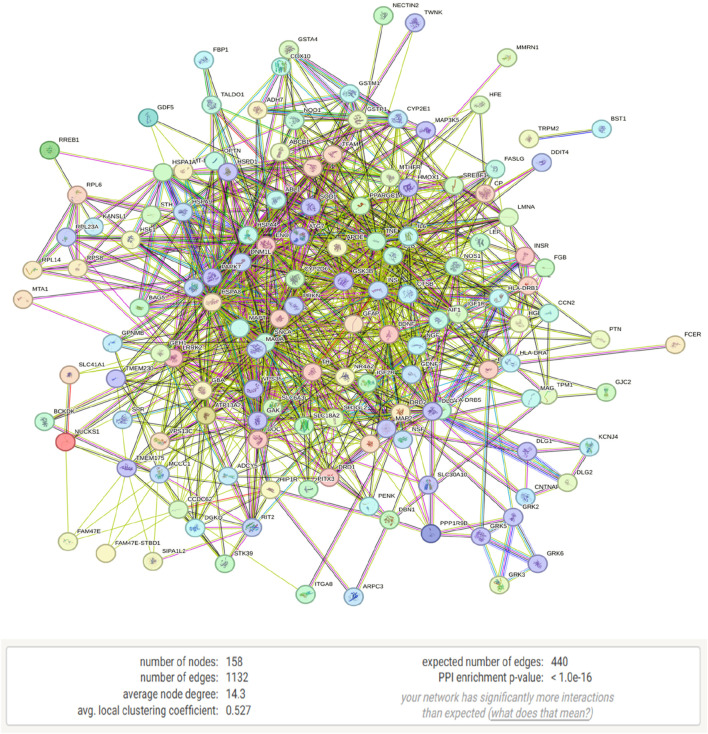
STRING analysis of Parkinson’s disease-related genes showing a protein-protein interaction (PPI) network consisting of 158 nodes and 1,132 edges.

**FIGURE 4:**
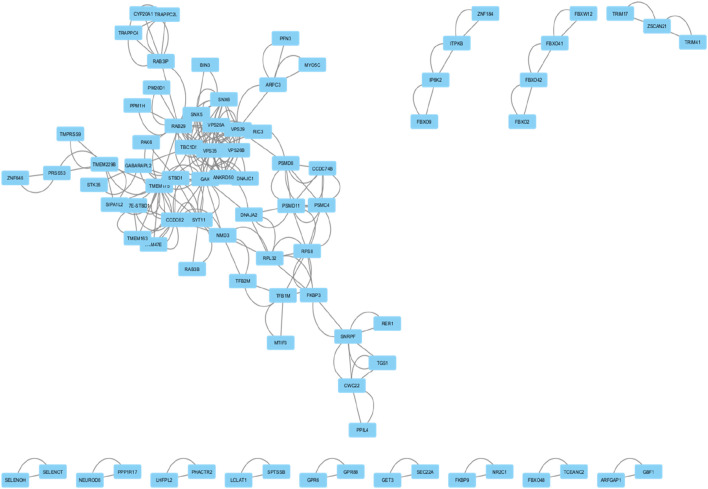
This network diagram illustrates the protein-protein interactions within a biological system. Nodes represent individual proteins, and edges indicate known interactions between them.

The selected genes were analyzed using the STRING database, revealing a protein-protein interaction (PPI) network in which 158 genes with 158 nodes (proteins) and 1,132 edges (interactions) shows connection and the left are disconnected and hidden. The PPI network exhibited a highly significant enrichment, with a p-value of <1.0e-16 and obtained from a random set of proteins which is a distinguishable property of STRING analysis, suggesting that these proteins are more interconnected than expected by chance, pointing to their potential roles in Parkinson’s disease pathology (Figures 3, 4). The bubble plot representing disease-gene associations and gene ontology-enriched biological pathways, with significance level is shown in Supplementary Figure 6-S2.

### Identification of hub genes

Using the CytoHubba plugin, ten hub genes with the highest interaction scores were identified within the PPI network. These genes, marked in red, demonstrated the highest degree of connectivity, making them strong candidates for further study in Parkinson’s disease. Some of these genes are also emerging as relevant to urinary biomarkers for Parkinson’s disease.

**FIGURE 5:**
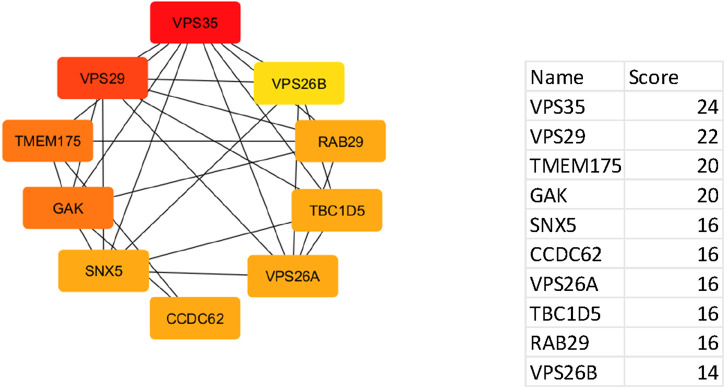
Network of top ten hub genes. The colour represents the degree of connectivity: red iindicates the highest degree of connectivity, orange indicates intermediate connectivity and yellow indicates low connectivity degree.

The top hub genes and their interaction scores are listed below:

These hub genes, particularly VPS35, VPS29, and GAK, hold potential not only as molecular drivers of Parkinson’s disease (Figure 5) but also as candidates for developing non-invasive urinary biomarkers for early diagnosis and therapeutic targeting.

## Discussion

The current systematic review demonstrated differentially expressed urine-based biomarkers in early and mid-stage IPD. Studies and systematic reviews on the identification of blood-based biomarkers for diagnosing PD have been published in 2022 (Chelliah et al., 2022). There is no compiled information till now on the studies demonstrating recognition of urinary metabolites in diagnosing PD. Seven urine metabolomics analysis studies in early IPD were identified in a database search. Characteristics of each metabolite found in these seven studies in terms of AUC, FC, MDA, and p-value have been described in detail in Supplementary Table S1. Few metabolites were identified to be related to the disease pathology. Three metabolites Acetylphenylalanine, (Luan et al., 2015a; Luan et al., 2015b; Kumari et al., 2020a), tyrosine (Luan et al., 2015a; Luan et al., 2015b; Kumari et al., 2020a) and Kynurenine, (Luan et al., 2015a; Bai et al., 2021; Luan et al., 2015b), were common in three studies. Furoglycine, Cortisol, Hydroxyphenylacetic acid, Glycine, Tiglyglycine, Aminobutyric acid, Hydroxyprogesterone, Phenylacetylglutamine, and Dihydrocortisol, were found common in two studies (Luan et al., 2015a; Luan et al., 2015b). Biopyrrin (Luan et al., 2015c), Homovanillic (Wichit et al., 2021), 5-Hydroxyindoleacetic acid (Wichit et al., 2021) and indole-3-acetic acid (Chung et al., 2023) are the metabolite which were found in three individual studies. The authors cautiously propose that elevated urinary indole-3-acetic acid (IAA) levels might serve as a potential biomarker for Parkinson’s disease (PD), but they emphasize the need for further confirmation through studies on larger cohorts and other biological samples to validate this finding (Chung et al., 2023). All metabolites showed dysregulation in the metabolic pathways. They all crossed the MDA and FC threshold indicating their ability to differentiate amongst three stages of PD and also to differentiate IPD from normal healthy subjects respectively. Our study produced a panel of altered urinary metabolites associated with idiopathic early and mid-stage PD.

### Role of acetyl phenylalanine in PD

Alanine derivatives were found elevated in early-stage PD indicating disrupted phenylalanine and tyrosine metabolism, which may be linked to PD patients’ reduced ability to synthesize dopamine (Luan et al., 2015a; Luan et al., 2015b; Luan et al., 2015c). There is a metabolite shift in the phenylalanine metabolism in PD from tyrosine production to producing trancinnamate instead (Shebl et al., 2024). This alteration may deprive the body of synthesizing dopamine, norepinephrine, and every other phenylalanine and tyrosine metabolite. Altered acetylphenylalanine levels cause phenylalanine metabolism to be disrupted in early-stage Parkinson’s disease and can be considered as a dopamine precursor (Ramdani et al., 2015), indicating its therapeutic role in the management of PD study have shown a statistically significant (p = 0.05) increase in acetyl phenylalanine in PD patients’ urine with fold change ranging from 1.2 to 2.5 between patients and controls (Sandler et al., 1969), and supposed to be correlated with various neurological conditions (Kobayashi et al., 1984).

### Role of Kynurenine in PD

This study suggested that urine KYN levels distinguished patients with early-stage PD and those with healthy controls. Urine KYN levels positively correlated with Hoehn-Yahr stages and disease durations, and negatively correlated with MMSE scores of patients with PD. These results suggested that urine KYN may represent a novel biomarker for detecting early-stage PD and evaluating the progression of PD. The urine KYN measurement might assist clinicians in identifying patients with early-stage PD (Bai et al., 2021). The kynurenine pathway has been linked to oxidative stress, immune system dysregulation, and excitotoxicity, all processes involved in PD pathology. Under the induction of pro-inflammatory cytokines, Kynurenine Pathway activation disrupts the balance between neuroprotection and neurotoxicity branches, tending to produce neurotoxic products 3-Hydroxyuric acid (a strong inducer of neurotoxic free radicals) and Quinolic acid, which play an important role in the pathogenesis of PD through excitotoxicity, oxidative stress, and/or inflammatory reactions (Chen and Geng, 2023). These changes in the pathology correspond to the early stage of PD. Neuroprotective metabolite kynurenic acid (KYNA) is reduced due to this alteration in the kynurenine pathway. KYNA analogs are important therapeutic agents in preventing or delaying PD.

### Role of tyrosine/hydroxyphenyl acetic acid in PD

Tyrosine is metabolized through several pathways, one of which leads to the production of hydroxyphenyl acetic acid. Hydroxyphenyl acetic acid is a downstream metabolite of tyrosine that results from its breakdown. In the early stages of Parkinson’s disease, the loss of dopamnergic neurons in the substantia nigra leads to reduced conversion of tyrosine into dopamine. As a result, the levels of tyrosine or its metabolites may become dysregulated. Studies suggest that alterations in tyrosine hydroxylase activity, the enzyme responsible for converting tyrosine to L-DOPA, may be detectable in early PD (Bogdanov et al., 2008; Hatano et al., 2016). A reduction in enzyme activity could indicate the early neurodegeneration process. Urinary and plasma metabolomic studies have identified elevated or altered levels of tyrosine and its metabolites in individuals with PD, which may occur even in the early stages of the disease. Tyrosine metabolism generates reactive oxygen species (ROS), contributing to oxidative stress, which is a key feature in the pathogenesis of PD (Fedorova et al., 2021).

Early research on the variance in steroidogenesis metabolism revealed that people with Parkinson’s disease (PD) had noticeably greater levels of cortisol in their saliva and blood (Charlett et al., 1998; Djamshidian et al., 2011; Hartmann et al., 1997). Stress-related cortisol release may be able to pass across the blood-brain barrier and bind to glucocorticoid receptors in the central nervous system. Cortisol excretion in the urine is thought to be a sign of elevated oxidative stress, which exacerbates dopamine cell degeneration in Parkinson’s disease (PD) (Joergensen et al., 2011).

Isoleucine and leucine levels in PD patients’ urine are strongly linked with their stage of the disease. Tremors, twitching, and muscular atrophy may be caused by a deficiency in leucine and isoleucine (Mally et al., 1997; Molina et al., 1997). Succinate functions as a junction point for multiple metabolic pathways and is involved in the production and removal of reactive oxygen species (Tretter et al., 2016).

Similar metabolites were found elevated in serum and saliva samples in PD patients (Kumari et al., 2020b). Patients with Parkinson’s disease have been shown to have higher concentrations of alanine, acetate, and trimethylamine N-oxide in both their serum and saliva samples (Kumari et al., 2020b; Kumari et al., 2020c). While alanine, acetate, and trimethylamine N-oxide content were considerably higher in both serum and saliva samples in patients with Parkinson’s disease (PD), phenylalanine, citrate, glutamine, and isoleucine were shown to be differentially raised in urine and saliva samples (Kumari et al., 2020b; Babu et al., 2018).

The PPI network for the selected 158 genes also exhibited highly significant connection with the identified urinary biomarkers. Ten hub genes with the highest interaction scores were identified within the PPI network. These genes, particularly VPS35, VPS29, and GAK, are central regulators of metabolic pathways that influence PD progression. For instance, genes such as VPS35 and VPS29, which regulate protein trafficking and degradation, may influence the levels of excreted biomolecules detectable in urine. Similarly, GAK and RAB29 are implicated in mitochondrial dysfunction, which has been linked to urinary biomarker research in Parkinson’s disease. The strong correlation between these genetic hub genes and urinary biomarkers suggests a genetic basis for the observed metabolic alterations in PD which is a novel finding. The review is one of the knowledge-enhancing chapters with few limitations. This review is conducted on early and mid-stage IPD patients’ controlled studies, hence making it less applicable to advanced stages of PD. Also, this review studied only urine based biomarkers, although urine sampling and analysis is more convenient than other methods of analysis. Small sample size and the incorporation of 3 studies (Hemi Luan et al.) by the same author leading to possibility of population overlapping and biased results are few limitations of this study. Although, two of the studies (Luan et al., 2015b; Luan et al., 2015c) had same patient population but not acknowledged anywhere in text. Moreover, they used different methods for sample analysis. As identification of metabolomics and their interaction with drug compounds has been recognised as a crucial part of drug repurposing. So, various advanced models like Graph CPIs, Graph Contrasting learning, Regular Wave Graph and heterogenous information network learning model can be used in future research to address the clinical utility of identified biomarkers, and to explore these gene-metabolite interactions to develop targeted therapies, thereby improving early detection and disease management strategies for PD (Zhao et al., 2025; Zhao et al., 2024; Cui et al., 2025; Chen et al., 2023).

## Conclusion

Our study found a total of 40 urinary biomarkers associated with idiopathic early-onset PD. These metabolites are involved in key metabolic pathways, such as phenylalanine and tryptophan metabolism, fatty acid β-oxidation, and neurotransmitter synthesis, all of which were found to be dysregulated in PD patients. Importantly, a set of core metabolites including acetyl phenylalanine, kynurenine, and tyrosine (metabolises to hydroxyphenyl acetic acid) were found to be consistently altered across multiple studies, further supporting their role as reliable PD biomarkers. Dysregulation in the genes can contribute to the altered metabolite profiles, providing insight into the mechanistic links between genetic variations and PD-related metabolic changes. Understanding these gene-metabolite relationships opens up new avenues for personalized medicine approaches, where genetic and metabolomic profiling could guide more tailored therapeutic strategies for PD patients. In conclusion, our findings support the use of urine-based metabolomics as a promising diagnostic tool in PD, while also demonstrating the significant genetic underpinnings of the metabolic alterations observed.

## Data Availability

The original contributions presented in the study are included in the article/Supplementary Material, further inquiries can be directed to the corresponding author.
