# Supplementary material for: Urinary based biomarkers identification and genetic profiling in Parkinson’s disease: a systematic review of metabolomic studies
Source: Front Bioinform. 2025 Mar 10;5:1513790. doi: 10.3389/fbinf.2025.1513790 (PMC11931117; doi:10.3389/fbinf.2025.1513790)
Supplement: Supplementary file 1 [file Table1.docx]

**Table S1 (supplementary material)**

**Summary of urinary metabolites between idiopathic PD patients and normal subjects**

|  |  |  |  | Early stage PD versus control subjects | | | | | | | | | | | | |
| --- | --- | --- | --- | --- | --- | --- | --- | --- | --- | --- | --- | --- | --- | --- | --- | --- |
|  |  | Wichit P et al (2021) | Chung S H et al (2023) | Hemi Luan et al (2015)^6^ | | | | Hemi Luan et al (2015)^11^ | | | | Hemi Luan et al (2015)^13^ | | Jia-he Bai et al (2020)^8^ | S. Kumari et al (2020)^14^ | |
| S. No. | METABOLITES | P value | P value | MDA | Fold change | AUC | p-value | VIP | P value | Fold change | AUC | VIP | AUC | AUC | AUC | P value |
| 1 | Indole-3-acetic acid |  | 0.03 |  |  |  |  |  |  |  |  |  |  |  |  |  |
| 2 | Homivanillic acid | 0.001 |  |  |  |  |  |  |  |  |  |  |  |  |  |  |
| 3 | 5-hydrxyindoleacetic acid | 0.001 |  |  |  |  |  |  |  |  |  |  |  |  |  |  |
| 2 | Biopyrrin |  |  |  |  |  |  |  |  |  |  | 2.25 with p<0.001 | 0.95 |  |  |  |
|  |  |  |  |  |  |  |  |  |  |  |  |  | 0.98 |  |  |  |
| 3 | Acetylephenylalanine^*LC^ |  |  | 2.68 | 3.21 | 0.88 | 9.28 × 10−16 | 3.00 | 3.06E-06 | 3.69 | 0.90 |  | |  | 0.645 | 0.0010 |
|  |  |  |  | 1.46 | 3.65 | 0.86 | 5.96 × 10−15 |  | 1.13E-16 | 6.51 | 0.93 |  |  |  |  |  |
| 4 | hydroxytryptophan^*LC^ |  |  | 0.00 | 2.62 | 0.78 | 9.19 × 10−11 | 1.96 | 3.17E-04 | 2.35 | 0.81 |  | |  |  |  |
|  |  |  |  | 1.63 | 3.05 | 0.86 | 5.18 × 10−14 |  | 6.22E-12 | 3.29 | 0.86 |  |  |  |  |  |
| 5 | kynurenine^*LC^ |  |  | 1.05 | 3.15 | 0.82 | 2.15 × 10−16 | 1.92 | 5.66E-04 | 3.44 | 0.80 |  | | 0.776 with p-value 0.000 |  |  |
|  |  |  |  | 2.13 | 3.27 | 0.84 | 1.39 × 10−13 |  | 2.16E-11 | 10.59 | 0.85 |  |  |  |  |  |
| 6 | Furoglycine^*LC^ |  |  |  | 3.28 | 0.80 | 1.50 × 10−15 | 1.92 | 2.23E-04 | 2.77 | 0.82 |  | |  |  |  |
|  |  |  |  | 1.54 | 4.34 | 0.86 | 9.28 × 10−17 |  | 3.42E-08 | 3.91 | 0.79 |  |  |  |  |  |
| 7 | Cortisol^*LC^ |  |  | 2.94 | 2.23 | 0.83 | 1.27 × 10−12 | 1.86 | 7.47E-05 | 2.43 | 0.84 |  | |  |  |  |
|  |  |  |  | 0.68 | 2.01 | 0.80 | 1.86 × 10−10 |  | 7.55E-11 | 2.35 | 0.84 |  |  |  |  |  |
| 8 | Hydroxyphenylacetic acid^*LC^ |  |  | 1.84 | 2.16 | 0.79 | 4.21 × 10−11 | 1.85 | 4.28E-03 | 1.66 | 0.75 |  | |  |  |  |
|  |  |  |  | 1.41 | 2.08 | 0.76 | 5.26 × 10−10 |  | 3.34E-09 | 2.78 | 0.81 |  |  |  |  |  |
| 9 | Glycine^*GC^ |  |  | 2.32 | 2.74 | 0.78 | 1.04 × 10−8 | 1.76 | 1.62E-03 | 4.48 | 0.77 |  | |  |  |  |
|  |  |  |  | 1.00 | 1.97 | 0.71 | 2.51 × 10−5 |  | 5.23E-11 | 6.70 | 0.84 |  |  |  |  |  |
| 10 | Tiglyglycine^*LC^ |  |  | 2.95 | 2.18 | 0.82 | 1.16 × 10−12 | 1.63 | 3.08E-03 | 2.13 | 0.75 |  | |  |  |  |
|  |  |  |  | 0.69 | 1.89 | 0.85 | 3.56 × 10−10 |  | 1.78E-10 | 2.54 | 0.83 |  |  |  |  |  |
| 11 | Aminobutyric acid^*GC^ |  |  | 1.93 | 3.67 | 0.78 | 1.46 × 10−9 | 1.47 | 7.15E-03 | 2.08 | 0.73 |  | |  |  |  |
|  |  |  |  | 3.00 | 4.71 | 0.79 | 1.93 × 10−11 |  | 1.07E-08 | 2.79 | 0.80 |  |  |  |  |  |
| 12 | Hydroxybenzoic acid^*LC^ |  |  |  |  |  |  | 1.47 | 3.08E-03 | 4.30 | 0.75 |  | |  |  |  |
|  |  |  |  |  |  |  |  |  | 3.08E-05 | 4.26 | 0.72 |  |  |  |  |  |
| 13 | Xanthurenic acid^*LC^ |  |  |  |  |  |  | 1.44 | 3.49E-03 | 2.01 | 0.75 |  | |  |  |  |
|  |  |  |  |  |  |  |  |  | 5.37E-08 | 2.25 | 0.78 |  |  |  |  |  |
| 14 | Hydroxyprogesterone^*LC^ |  |  | 2.01 | 2.23 | 0.83 | 7.99 × 10−11 | 1.43 | 1.08E-04 | 1.83 | 0.83 |  | |  |  |  |
|  |  |  |  | 1.85 | 2.52 | 0.84 | 4.43 × 10−12 |  | 1.10E-09 | 2.04 | 0.82 |  |  |  |  |  |
| 15 | Isoleucine^*GC^ |  |  |  |  |  |  | 1.4 | 3.25E-02 | 1.94 | 0.68 |  | |  | 0.647 | 0.0022 |
|  |  |  |  |  |  |  |  |  | 4.95E-09 | 2.77 | 0.80 |  |  |  |  |  |
| 16 | Alanine^*GC^ |  |  |  |  |  |  | 1.34 | 1.39E-02 | 1.94 | 0.71 |  | |  |  |  |
|  |  |  |  |  |  |  |  |  | 6.35E-08 | 2.68 | 0.78 |  |  |  |  |  |
| 17 | Leucine^*GC^ |  |  |  |  |  |  | 1.27 | 4.19E-02 | 1.39 | 0.67 |  | |  |  |  |
|  |  |  |  |  |  |  |  |  | 3.34E-07 | 2.46 | 0.77 |  |  |  |  |  |
| 18 | Phenylacetylglutamine^*LC^ |  |  | 2.07 | 2.02 | 0.78 | 2.79 × 10−9 | 1.25 | 4.46E-04 | 1.79 | 0.80 |  | |  |  |  |
|  |  |  |  | 2.02 | 2.25 | 0.78 | 3.61 × 10−10 |  | 2.30E-07 | 2.06 | 0.77 |  |  |  |  |  |
| 19 | Dihydrocortisol^*LC^ |  |  |  |  |  |  | 1.24 | 2.72E-03 | 1.55 | 0.76 |  | |  |  |  |
|  |  |  |  |  |  |  |  |  | 5.84E-08 | 1.72 | 0.78 |  |  |  |  |  |
| 20 | Phenylacetic acid |  |  | 3.49 | 2.36 | 0.86 | 1.50 × 10−15 |  |  |  |  |  | |  |  |  |
|  |  |  |  | 3.12 | 2.71 | 0.87 | 9.28 × 10−17 |  |  |  |  |  |  |  |  |  |
| 21 | Tryptamine |  |  | 2.57 | 2.74 | 0.83 | 2.26 × 10−14 |  |  |  |  |  | |  |  |  |
|  |  |  |  | 3.54 | 3.31 | 0.85 | 2.68 × 10−16 |  |  |  |  |  |  |  |  |  |
| 22 | Indolelactic acid |  |  | 3.23 | 3.36 | 0.84 | 2.15 × 10−16 |  |  |  |  |  | |  |  |  |
|  |  |  |  | 1.66 | 3.31 | 0.81 | 1.39 × 10−13 |  |  |  |  |  |  |  |  |  |
| 23 | Hydroxyphenylacetylglycine |  |  | 2.00 | 2.08 | 0.84 | 4.88 × 10−12 |  |  |  |  |  | |  |  |  |
|  |  |  |  | 1.79 | 2.76 | 0.86 | 2.16 × 10−14 |  |  |  |  |  |  |  |  |  |
| 24 | Phenylacetylglycine |  |  | 1.75 | 2.20 | 0.81 | 1.50 × 10−15 |  |  |  |  |  | |  |  |  |
|  |  |  |  | 2.55 | 2.96 | 0.85 | 9.28 × 10−17 |  |  |  |  |  |  |  |  |  |
| 25 | Furoylglycine |  |  | 0.00 | 3.28 | 0.80 | 1.50 × 10−15 |  |  |  |  |  | |  |  |  |
|  |  |  |  | 1.54 | 4.34 | 0.86 | 9.28 × 10−17 |  |  |  |  |  |  |  |  |  |
| 26 | Glutamyltyrosine |  |  | 3.18 | 16.86 | 0.84 | 1.41 × 10−11 |  |  |  |  |  | |  | 0.619 | 0.0065 |
|  |  |  |  | 3.38 | 17.0 | 0.86 | 5.22 × 10−15 |  |  |  |  |  |  |  |  |  |
| 27 | Acetyltyrocine |  |  | 1,93 | 2.45 | 0.83 | 2.89 × 10−13 |  |  |  |  |  | |  |  |  |
|  |  |  |  | 1.67 | 2.85 | 0.86 | 4.95 × 10−15 |  |  |  |  |  |  |  |  |  |
| 28 | 5,6-dihydroxyindole |  |  | 1.88 | 2.17 | 0.82 | 9.99 × 10−11 |  |  |  |  |  | |  |  |  |
|  |  |  |  | 0.94 | 2.48 | 0.86 | 3.69 × 10−13 |  |  |  |  |  |  |  |  |  |
| 29 | Deoxyinosine |  |  | 0.00 | 2.16 | 0.72 | 3.53 × 10−7 |  |  |  |  |  | |  |  |  |
|  |  |  |  | 0.81 | 2.44 | 0.77 | 1.00 × 10−9 |  |  |  |  |  |  |  |  |  |
| 30 | Malonylcarnitine |  |  | 0.73 | 2.02 | 0.80 | 1.68 × 10−10 |  |  |  |  |  | |  |  |  |
|  |  |  |  | 1.00 | 2.32 | 0.79 | 7.09 × 10−11 |  |  |  |  |  |  |  |  |  |
| 31 | Urocanic acid |  |  | 1.96 | 2.06 | 0.80 | 2.94 × 10−10 |  |  |  |  |  | |  |  |  |
|  |  |  |  | 1.00 | 2.25 | 0.80 | 5.59 × 10−11 |  |  |  |  |  |  |  |  |  |
| 32 | Spermidine |  |  | 0.73 | 2.77 | 0.72 | 4.05 × 10−9 |  |  |  |  |  | |  |  |  |
|  |  |  |  | 0.62 | 2.67 | 0.72 | 2.53 × 10−8 |  |  |  |  |  |  |  |  |  |
| 33 | Naphthol |  |  | 1.40 | 2.67 | 0.82 | 7.60 × 10−10 |  |  |  |  |  | |  |  |  |
|  |  |  |  | 1.37 | 2.76 | 0.79 | 1.12 × 10−8 |  |  |  |  |  |  |  |  |  |
| 34 | Trimethylamine N-oxide |  |  | 0.78 | 2.37 | 0.83 | 3.40 × 10−10 |  |  |  |  |  | |  |  |  |
|  |  |  |  | 1.00 | 3.05 | 0.84 | 4.09 × 10−11 |  |  |  |  |  |  |  |  |  |
| 35 | Ornithine |  |  |  |  |  |  |  |  |  |  |  | |  | 0.778 | <0.01 |
| 36 | β- hydroxybutyrate |  |  |  |  |  |  |  |  |  |  |  | |  | 0.639 | 0.004 |
| 37 | Succinate |  |  |  |  |  |  |  |  |  |  |  | |  | 0.632 | 0.007 |
| 38 | Tyrosine/hydroxyphenyl acetic acid |  |  | 1.84 | 2.16 | 0.79 | 4.21 × 10−11 | 1.56 | 1.38E-01 | 2.18 | 0.63 |  | |  | 0.6129 | 0.006 |
|  |  |  |  | 1.41 | 2.08 | 0.76 | 5.26 × 10−10 |  | 9.09E-08 | 3.64 | 0.78 |  |  |  |  |  |

Metabolites proved by reference standards are indicated by (*). Metabolites discovered with GC-MS and LC-MS were indicated by superscript GC or LC, respectively.

The OPLS-DA model yielded the variable importance in the projection (VIP) with a threshold of 2.0. The Wilcoxon − Mann U test was used to get the P-value (<0.001). By contrasting the metabolites in the PD group with those in the control group, fold change (FC) was calculated. Area under the ROC curve (AUC). Values are mentioned in two rows for single metabolite in which upper row and lower row indicate values for early stage PD versus controls and mid-stage PD versus controls respectively.
