# Supplementary material for: Urinary based biomarkers identification and genetic profiling in Parkinson’s disease: a systematic review of metabolomic studies
Source: Front Bioinform. 2025 Mar 10;5:1513790. doi: 10.3389/fbinf.2025.1513790 (PMC11931117; doi:10.3389/fbinf.2025.1513790)

Figure 6 (S2) (supplementary material)

The bubble plot represents (A) disease-gene associations and (B) gene ontology-enriched biological pathways, where the colour intensity indicates the false recovery rate (FDR), with lighter colours showing stronger statistical significance. The bubble size corresponds to the number of genes associated with each pathway

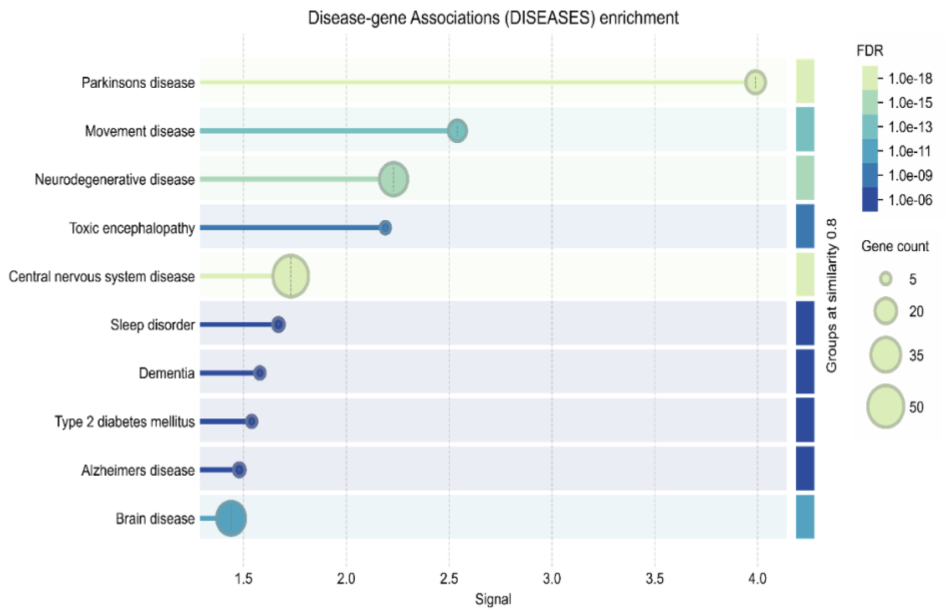

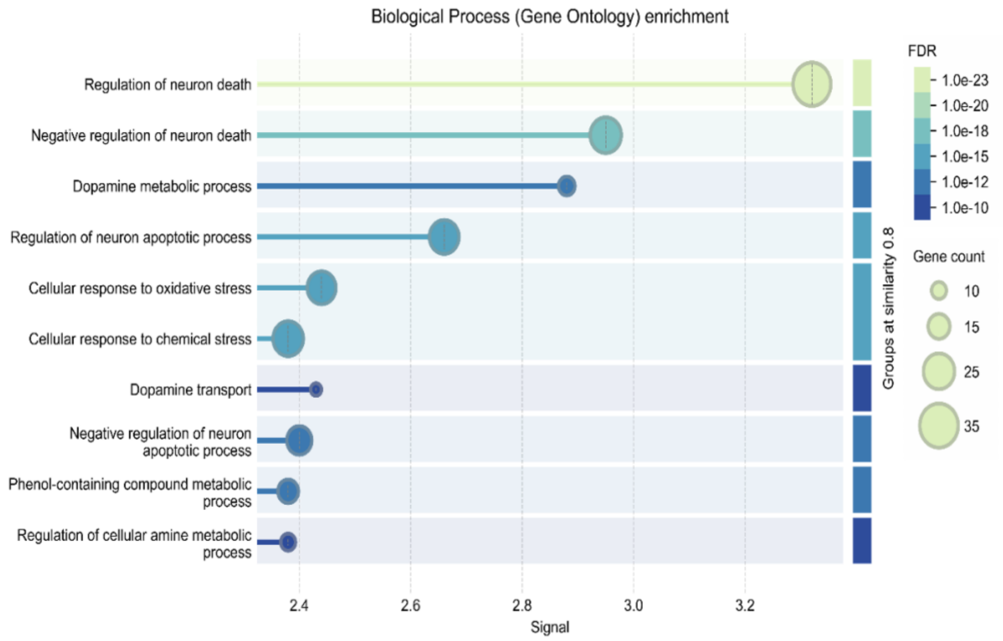

Supplement: Supplementary file 2 [file Image1.pdf]
